# Vancomycin-specific T Cell responses in allergic and non-allergic individuals

**DOI:** 10.1186/2045-7022-4-S3-O5

**Published:** 2014-07-18

**Authors:** Marta E Polak, Carolann McGuire, Alvin Lee, Ursula Quinn, Anthony Williams, Efrem Eren, Michael R Ardern-Jones

**Affiliations:** 1 Faculty of Medicine, University of Southampton, Southampton, UK

## Background

The association between specific HLA alleles and risk of severe cutaneous drug hypersensitivity reactions is increasingly recognised. Vancomycin mediated pseudo-allergic 'red man syndrome' is well recognised, yet urticaria/anaphylaxis is uncommon and some clinical reactions are delayed. Although this may suggest the presence of vancomycin initiated T cell responses, no predisposing vancomycin HLA association has been identified. Therefore we set out to examine the role of T cell responses in vancomycin hypersensitivity reactions.

## Methods

Of our tested cohort of 103 individuals who suffered delayed skin drug hypersensitivity reactions, vancomycin was deemed the culprit in 17, who developed drug exanthems (59%), DRESS (35%), or SJS/TEN (18%). We used ELISpot and [3H]-Thymidine assays to measure vancomycin-specific *ex-vivo* IFN-/ IL-4 and proliferation above background.

## Results

Individuals with drug hypersensitivity showed a mean circulating PBMC frequency of vancomycin-specific cells of 470.0 x10-4% (IFN-) / 33.5 x10-4% (IL-4) and SI 4.08 if tested within 30 days, whilst those tested after 30 days showed 316.0 x10-4% (IFN-) / 20.0 x10-4% (IL-4) and SI 3.43. Individuals never previously exposed to vancomycin (n=11) showed a lower mean circulating frequency: 2.1 x10-4% (IFN-, p<0.005)) / 1.7 x10-4% (IL-4, p=0.27) and SI 1.28. In controls who had been exposed to vancomycin (without any evidence of a hypersensitivity reaction, n=6) detectable frequencies were also lower than allergics: 11 x10-4% (IFN-) / 7.1 x10-4% (IL-4) and SI 0.98. We co-cultured non-allergic PBMC with vancomycin for two-weeks in vitro. In two lines, we saw expansion of vancomycin-specific T cells in ELISpot to an average frequency of 954 x10-4% (IFN-) and 1130 x10-4% (IL-4) which was confirmed by ELISpot and intracellular cytokine staining.

## Conclusion

These data confirm that robust Th1 responses target vancomycin in cutaneous hypersensitivity reactions, which supports the use of T cell inhibition such as steroids and ciclosporin. Vancomycin-specific responses appear reduced with time which advocates early diagnostic testing, but the clinical possibility of loss of sensitisation is intriguing. The finding that vancomycin-specific T cells can be artificially expanded from individuals without clinical reactivity, suggests that both immune predisposition and potentially adaptive regulation may be important in development of hypersensitivity responses to vancomycin.

